# Effect of intraocular anti-VEGF on cystoid macular edema associated with Henoch-Schonlein purpura-a case report

**DOI:** 10.1186/s12886-020-01351-x

**Published:** 2020-02-28

**Authors:** Dongyan Pan, Xiao Cui, Weiye Zhu, Haifeng Qin, Qing Li, Bing Xu, Jin-hui Wu

**Affiliations:** 1 Department of Ophthalmology, Changhai Hospital, Second Military Medical University, Shanghai, China; 2 Department of Ophthalmology, Third Hospital Affiliated to Second Military Medical University, Shanghai, China

**Keywords:** Henoch-Schonlein purpura, Macular edema, Anti-vascular endothelial growth factor

## Abstract

**Background:**

To report a bilateral cystoid macular edema associated with Henoch-Schonleinpurpura.

**Case presentation:**

A 21-year-old man presented a bilateral, painless, and bilateral blurred vision for 5 weeks with visual acuity (VA) of 6/12 on the right eye and 6/48 on the left. FA and OCT showed bilateral cystoid macular edema, and the fundus photograph showed retinal hemorrhages. Using intravenous dexamethasone could reduce macular edema, but it reoccurred shortly after switching to oral prednisone. Repeated intraocular injection of anti-VEGF in both eyes was performed and VA improved to 6/6 on the right eye and 6/7.5 on the left with the regression of edema after 6 months follow-up.

**Conclusions:**

Intraocular anti-VEGF might be an alternative choice to glucocorticoid in cases of bilateral cystoid macular edema associated with Henoch-Schonlein purpura.

## Background

Henoch-Schönlein purpura (HSP; also referred to as Schönlein-Henoch purpura, anaphylactoid purpura, or purpurarheumatica) is an acute disorder mediated by immunoglobulin A with a feature of a generalized vasculitis involving the small vessels of the skin, the gastrointestinal tract, the kidneys, the joints, and, rarely, the lungs and the central nervous system. Ocular involvement is rare. Here we report a case in which an HSP patient had bilateral cystoid macular edema with round-like retinal hemorrhages.With the treatment of systemic glucocorticoid and intraocular anti-vascular endothelial growth factor the macular edema remitted.

## Case presentation

A 21-year-old man presented a bilateral, painless, and blurred vision for 5 weeks. Before ocular symptoms, he had got a mild cold and cough for several days. Two years ago, he was diagnosed with HSP, manifesting symptoms of hematuria and purpura. His clinical manifestations were resolved with the treatment of intravenous methylprednisolone followed by oral prednisolone. He had only once recurrence of symptoms after an attack of influenza during the 2 years.

Five weeks ago, he experienced blurred vision in both eyes, especially in the left eye but he didn’t pay much attention. At that time, he was taking 2.5 mg oral prednisolone per day and he ceased prednisolone 1 week later. At present, the patient had no symptoms of active HSP symptoms and had normal blood pressure. He denied a history of hypertension and trauma. There were no abnormal findings upon the total blood cell count, coagulation profile, and the function of the liver and kidney.

**Fig. 1:**
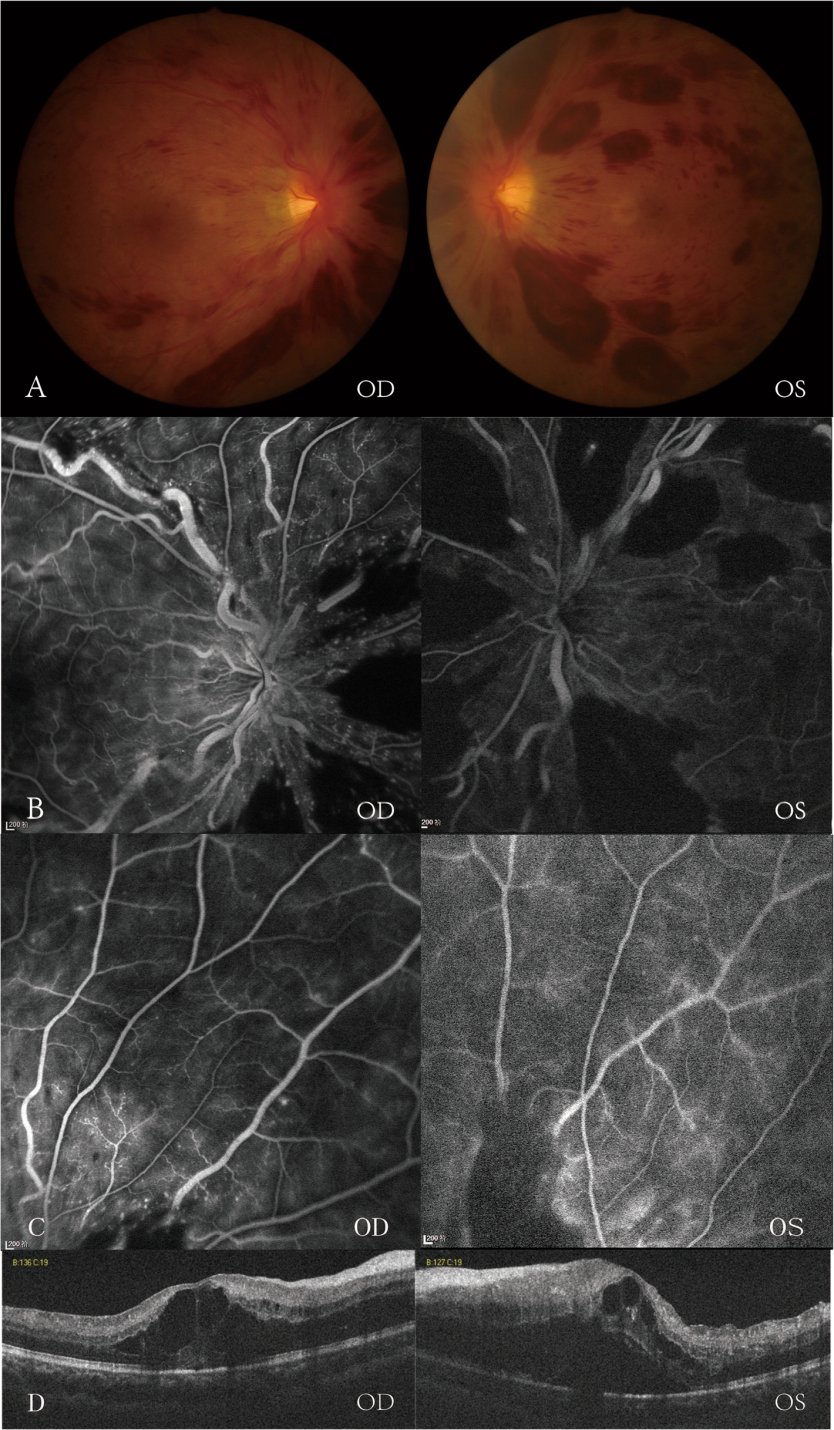
Fundus examination (**a**) and retinal fluorescein angiography(FA) (**b**, **c**) showed diffuse, tortuous, and dilated retinal vein with superficial flame-shaped retinal hemorrhages. Nonperfusion was not noticed in the FA images in the peripheral retina in both eyes. Optical coherence tomography (**d**) revealed cystoid macular edema in both eyes. The left eye was more severe than the right eye

On examination, his best-corrected visual acuity (BCVA) was 6/12 on the right eye and 6/48 on the left, with a relative afferent pupillary defect in the left eye. The anterior chamber was quiet. Fundus examination and retinal fluorescein angiography(FA) revealed diffuse, tortuous, and dilated retinal vein with scattered round-like retinal hemorrhages (Fig. 1a-c). The optical coherence tomography (OCT) showed cystoid macular edema (ME) in both eyes (Fig. 1d).

**Fig. 2:**
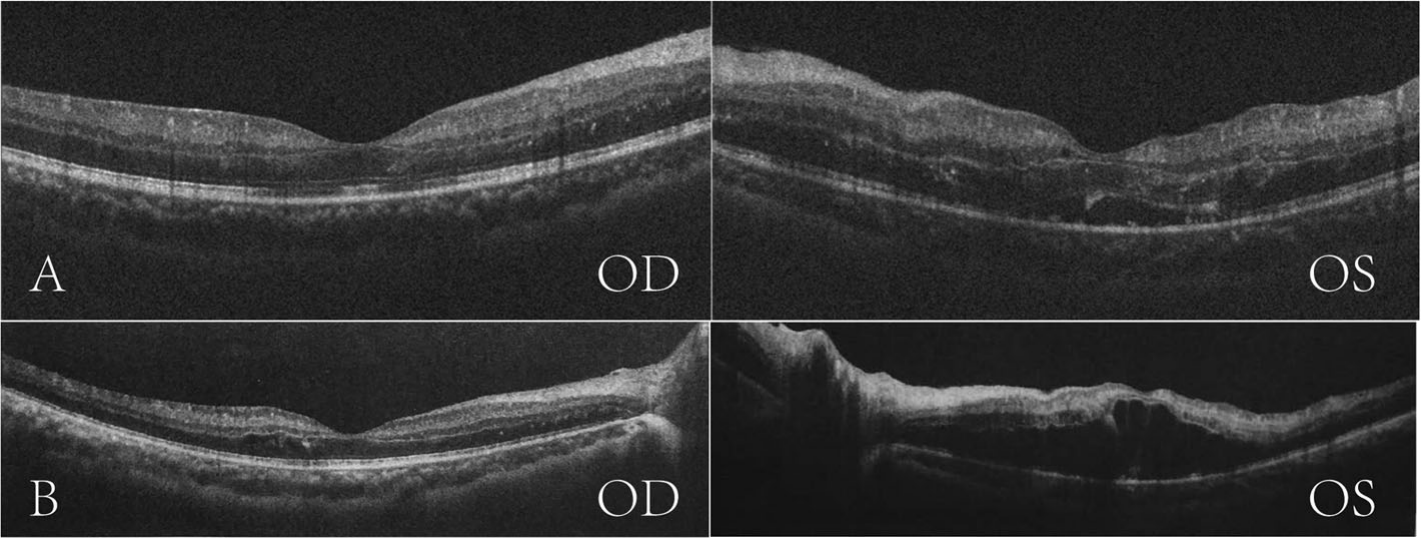
Optical coherence tomography revealed a significant reduction in macular edema in both eyes (**a**). Macular edema reoccurred after glucocorticoid tapering (**b**)

The patient began to take intravenous dexamethasone 10 mg per day and experienced a significant improvement on the following day. Seven days later, his BCVA was 6/6 on the right eye and 6/12 on the left with a significant reduction in macular edema, which was demonstrated by the OCT images (Fig. 2a).

**Fig. 3:**
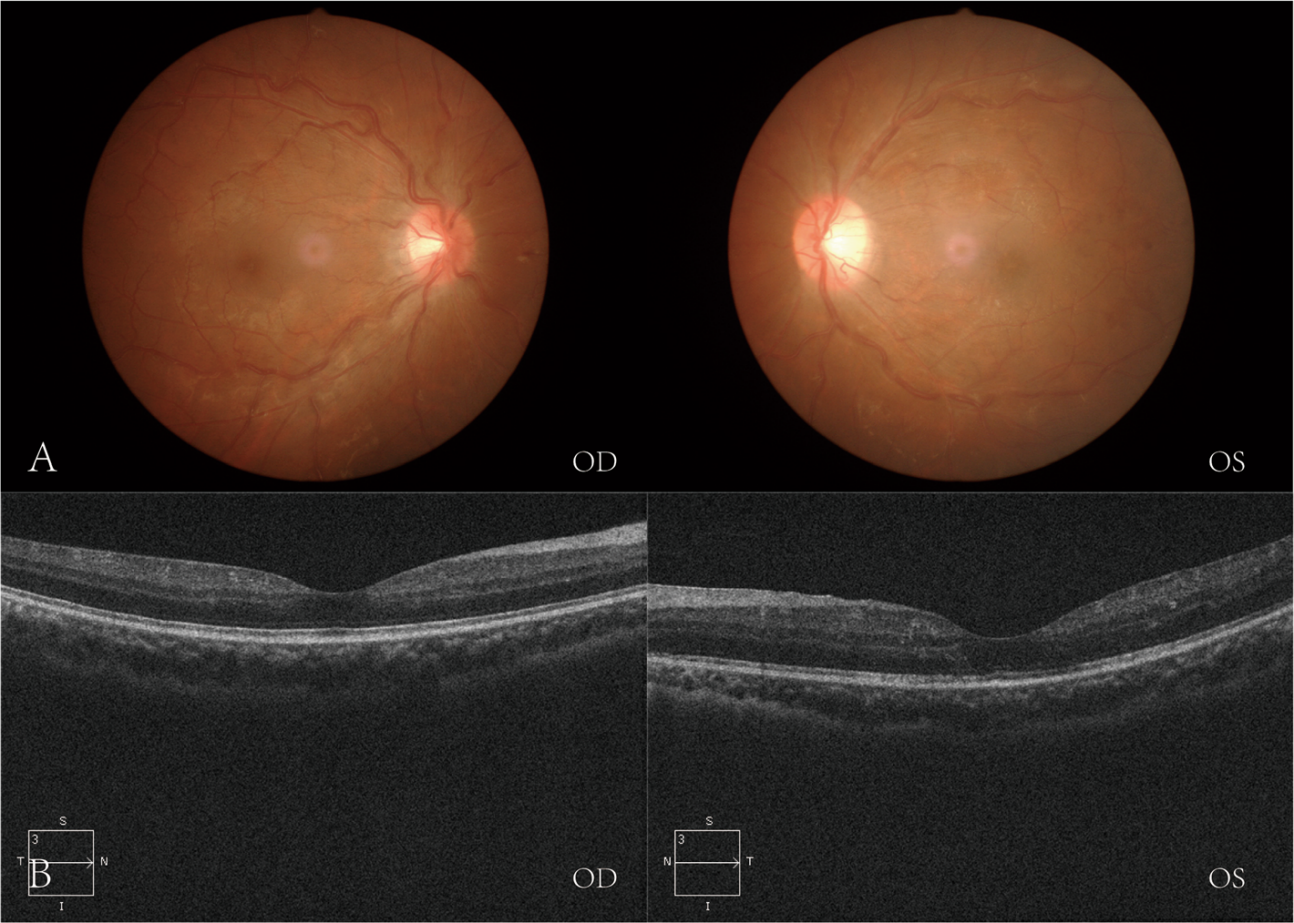
Regression of retinal hemorrhages (**a**) and macular edema (**b**) after intraocular injection of anti-VEGF in both eyes

**Fig. 4:**
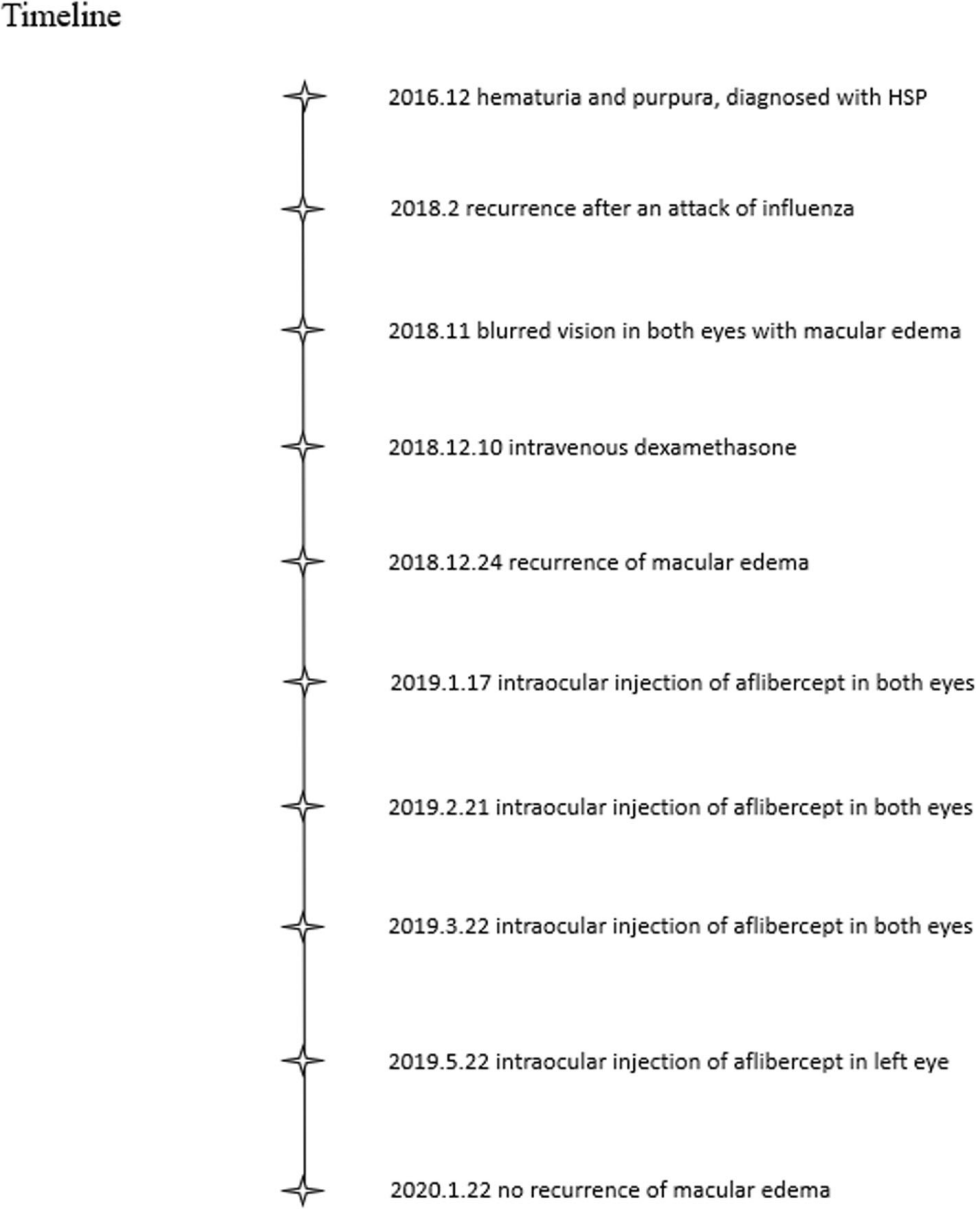
Timeline of the case

Unfortunately, ME of the left eye reoccurred 1 week after he switched to oral prednisoneME (Fig. 2b), and the vision decreased to 6/60 again on the left eye and remained 6/6 on the right eye. He received *3 + PRN* of *intravitreal* aflibercept injection in both eyes, with three injections in the right eye and four injections in the left eye. His VA improved to 6/6 on the right eye and 6/7.5 on the left with the regression of retinal hemorrhages (Fig. 3a) and then faded awayfrom macular edema (Fig. 3b) after 6 months follow-up (Fig. 4).

## Discussions and conclusion

HSP is one of the most common types of vasculitis in children which rarely presents in adults. Reported HSP cases with ocular involvement includes keratitis and granulomatous uveitis [1, 2]; unilateral anterior ischemic optic neuropathy [3]; bilateral central retinal artery occlusion [4]; and subperiosteal orbital hematomas [5]. Bilateral cystoid macular edema and cotton wool spots were previously reported in two patients with Henoch-Schonlein purpura [6, 7]. Both of the two patients had high blood pressure and minimal retinal hemorrhages. Their conditions improved after steroid therapy. While in our case, the patient presented with normal blood pressure without any history of hypertension. Besides, he had more severe hemorrhage with no cotton wool spots. Macular edema *subsided* with steroid therapy but relapsed shortly after glucocorticoid tapering.

Vascular endothelial growth factor (VEGF)is a highly specific mitogen for vascular endothelial cells. In response to hypoxia, the expression of VEGF is upregulated by activatingoncogenes and a variety of cytokines. VEGF is also known to play an essential role in breaking down the blood-retinal barrier. VEGF induces the related inflammatory cytokines via VEGF receptors [8]. Therefore, the anti-VEGF agent could reduce the related inflammatory cytokines indirectly, resulting in a decrease of inflammation. However compared with anti-VEGF therapy, the reduction of these cytokines is greater in effect of steroids. The possible role of VEGF in the pathogenesis of HSP was discussed in the previous studies [9–11]. They found that VEGF levels in plasma were significantly higher during both the acute phase and the resolution phase compared to healthy control groups. Compared with acute vasculitic lesions, the VEGF expression in the epidermis and the vascular bed was more intense in resolving lesions [11]. The results suggested that as a potent permeability, chemotactic, and migratory factor, VEGF might play a crucial role in the morphological and functional changes of the vascular bed and inflammatory reaction in HSP. In our case, the patient was in resolution phase so there was a possibly high level of VEGF in the retina which induced the pathological changes and provided a theoretical basis for anti-VEGF therapy. Although anti-VEGF therapy is widely used in the treatment of diabetic macular edema, neovascular macular degeneration, choroidal neovascularization secondary to pathologic myopia and macular edema due to retinal vein occlusion, anti-VEGF therapy for macular edema associated with Henoch-Schonlein purpura is rare. More researches are needed to confirm its therapeutic value in such kind of patients.

In this case, the left eye of our patient had more retinal hemorrhage and more severe macular edema than the right eye. Muraoka et al. reported that the mean size of maximum peripheral dot or blot hemorrhage was larger in eyes which were classified as ischemic comparing to the eyes classified as nonischemic [12]. Although nonperfusion was not noticed in the FA images of the patient’s eyes, the left eye received more injections than the right eye. It is possible that macular edema might recur even after 3 + PRN treatment of intravitreal aflibercept injections due to a high level of VEGF. Even though macular edema *hasn’t* recurred *6 months after the last injection, there is still a need for further* clinical *follow*-*up.*

In conclusion, although glucocorticoid therapy was useful for cystoid macular edema in this case, there was a reoccurrence after corticosterone reduction. In such situation, intraocular anti-VEGF might be an alternative choice.

## Data Availability

All data generated or analyzed during this study are included in this published article.

## Abbreviations

BCVA: Best-corrected visual acuity
HSP: Henoch -Schönlein purpura
OCT: Optical coherence tomography
VEGF: Vascular endothelial growth factor
